# How beta diversity and the underlying causes vary with sampling scales in the Changbai mountain forests

**DOI:** 10.1002/ece3.3493

**Published:** 2017-10-24

**Authors:** Lingzhao Tan, Chunyu Fan, Chunyu Zhang, Klaus von Gadow, Xiuhua Fan

**Affiliations:** ^1^ The Key Laboratory for Forest Resources and Ecosystem Processes of Beijing Beijing Forestry University Beijing China; ^2^ Georg‐August Universität Göttingen Göttingen Germany; ^3^ Department of Forestry and Wood Science University of Stellenbosch Stellenbosch South Africa; ^4^ The School of Science Beijing Forestry University Beijing China

**Keywords:** beta diversity, dispersal limitation, environmental filtering, scale effects, variation partitioning

## Abstract

This study aims to establish a relationship between the sampling scale and tree species beta diversity temperate forests and to identify the underlying causes of beta diversity at different sampling scales. The data were obtained from three large observational study areas in the Changbai mountain region in northeastern China. All trees with a dbh ≥1 cm were stem‐mapped and measured. The beta diversity was calculated for four different grain sizes, and the associated variances were partitioned into components explained by environmental and spatial variables to determine the contributions of environmental filtering and dispersal limitation to beta diversity. The results showed that both beta diversity and the causes of beta diversity were dependent on the sampling scale. Beta diversity decreased with increasing scales. The best‐explained beta diversity variation was up to about 60% which was discovered in the secondary conifer and broad‐leaved mixed forest (CBF) study area at the 40 × 40 m scale. The variation partitioning result indicated that environmental filtering showed greater effects at bigger grain sizes, while dispersal limitation was found to be more important at smaller grain sizes. What is more, the result showed an increasing explanatory ability of environmental effects with increasing sampling grains but no clearly trend of spatial effects. The study emphasized that the underlying causes of beta diversity variation may be quite different within the same region depending on varying sampling scales. Therefore, scale effects should be taken into account in future studies on beta diversity, which is critical in identifying different relative importance of spatial and environmental drivers on species composition variation.

## 1 INTRODUCTION

Beta (β) diversity, a subject of interest to community ecologists, which is introduced by Whittaker (1960) as the ratio between regional (γ) and local species diversity (α), has also been developed as the variation in species composition among sites within a geographical area of interest (Anderson et al., 2011; Gorelick, 2011), or to describe the species turnover from site to site (Anderson et al., 2011). Beta diversity reflects the spatial organization of species diversity. As a consequence, it provides fundamental insights into community assembly.

There is much agreement that beta diversity depends on the spatial scales (Barton et al., 2013; De Cáceres et al., 2012; Palmer & White, 1994; Steinbauer, Dolos, Reineking, & Beierkuhnlein, 2012). Ecologists typically measure the scale in terms of extent and grain (Nekola & White, 1999; Whittaker, Willis, & Field, 2001). A framework has summarized priori expectations for how beta diversity might vary among sampling grains drawn at the spatial extent ranging from local, regional to global scales (Barton et al., 2013). Generally, if the sampling grain is fixed and spatial extent is allowed to increase, beta diversity will naturally increase monotonically. Because when new sampling grains are incorporated, variation in stochastic occupancy patterns among sites and deterministic variation in species responses to habitat heterogeneity would result in increasing compositional dissimilarities between sampling grains. However, if spatial extent is fixed and sampling grain is allowed to vary, beta diversity might decrease monotonically. Because larger sampling grains can capture a larger portion of the community, and similarity between sampling units would therefore increase (Barton et al., 2013).

Theoretically, the variation of beta diversity across different sampling grains may be the result of different processes operating at various spatial scales (De Cáceres et al., 2012; Laliberté, Paquette, Legendre, & Bouchard, 2009). At local scales (<10^6^ m^2^), environmental filtering and dispersal limitation have been suggested to the main deterministic processes that contribute to beta diversity. Environmental filtering assumes that environmental conditions and species niche preferences control species composition. Sampling units with similar habitats are more likely to have similar species compositions (Kraft et al., 2011; Kristiansen et al., 2012). Dispersal limitation suggests that species composition may be determined by spatial process, which emphasizes the effects of spatial dispersal history. Under dispersal limitation, sampling units that are located in close proximity to each other should have a more similar species composition than units that are distant from each other (Chisholm & Lichstein, 2009; Qiao et al., 2015). Biotic interactions and stochastic process also contribute to beta diversity, but those processes are difficult to be quantified as far as now. At regional and global scale, ecological and evolutionary processes will also affect species composition of local communities. Those processes, including speciation, extinction, or biogeographic dispersal, should be taken into account when comparing beta diversity across regional or global scales (Whittaker et al., 2001). As a result, the spatial variation in species compositions is of particular interest to ecologists when determining what processes generate and maintain biodiversity in ecosystems (Chase & Myers, 2011; Kraft et al., 2011; Leenheer, Narang, Lewis, & Atwater, 2002; Legendre & De Cáceres, 2013; Tuomisto, Ruokolainen, & Ylihalla, 2003).

At present, beta diversity has been studied in a great variety of forest ecosystems, including temperate and tropical forests (Myers et al., 2013), Amazonian palm communities (Kristiansen et al., 2012), Southern African dry woodlands (De Cauwer, Geldenhuys, Aerts, Kabajani, & Muys, 2016), and subtropical broad‐leaved forests in China (Legendre et al., 2009; Qiao et al., 2015). While unfortunately, even though some studies have reported the variation of beta diversity across large spatial extent (Kraft et al., 2011; Lenoir et al., 2010; Qian & Ricklefs, 2007), limited studies have specifically investigated the scale dependence of beta diversity and how the processes driving beta diversity variation vary across different spatial scales of sampling grains (Barton et al., 2013). The establishment of large permanent forest plots makes it possible to compare beta diversity values and the underlying local ecological processes at different sampling grains. However, to our knowledge, only few studies investigated how the causes of beta diversity variation depend on sampling grains (De Cáceres et al., 2012; Laliberté et al., 2009). The patterns and causes of scaling beta diversity need more evidence to be thoroughly explored.

In this study, beta diversity was calculated as the total variance of community data. To identify the causes of beta diversity variation, variation partitioning analysis was used to determine the contribution of environmental variables and spatial variables. To test the scale effects, beta diversity and the relative importance of environmental and spatial processes were assessed for four different sampling grains, ranging from 20 × 20 m to 50 × 50 m. The objective is to understand the underlying causes of beta diversity variations across sampling grains, based on two hypotheses:

- The beta diversity values of tree species decreases with increasing sampling grains when the spatial extent of sampling area is fixed;
- The causes underlying beta diversity are varying across different spatial grains.

## 2 MATERIALS AND METHODS

### 2.1 Study area

**Figure 1:**
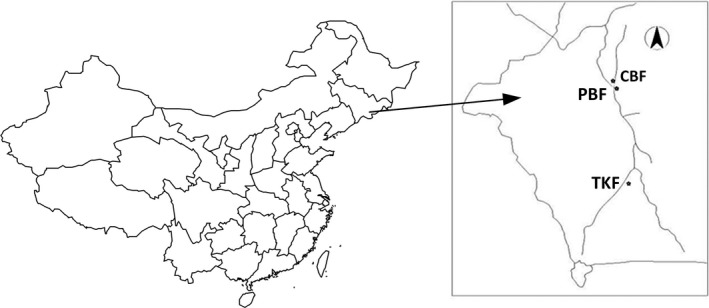
Location of the three observational field studies, measuring 260 × 200 m each in Changbai Mountains in northeastern China

This study was conducted at three observational study plots in the Changbaishan Nature Reserve, located in northeastern China (Figure 1) at 41°41′–42°25′N and 127°42′–128°17′E (Chinese Forests Editorial Committee 1999; Zheng, Jiang, Zeng, & DU, 2004). The plots represent three distinct forest communities at different altitudes and varying in species richness.

The Changbai mountains are situated in a temperate monsoon montane climate, characterized by both continental weather conditions and a temperate monsoon. The observational study was established at the Changbaishan Forest Ecosystems Research Station, which has an annual mean temperature of 3.6°C. The average coldest monthly temperature is −15.4°C in January, and the average hottest one is 19.6°C in July. The mean annual precipitation is about 695 mm, concentrated during the period from June to September. The mean relative air humidity is around 72% (Zhang et al., 2005). The soil properties at the Changbaishan Nature Reserve differ along an elevational gradient. Montane tundra soils dominate at the highest elevations, changing to montane forest sward soils and montane brown coniferous forest soils at midelevations, and finally, dark brown forest soils at the lowest elevations. The rootable depths range from 10 to 130 cm.

The broad‐leaved Korean pine forest is the most frequent forest type. Major tree species include deciduous broad‐leaved species such as *Tilia amurensis, Quercus mongolica, Betula ermanii*,* Populus ussuriensis,* and *Fraxinus mandschurica*. The dominant conifer species are *Pinus koraiensis, Abies fabri, Abies holophylla,* and *Picea jezoensis*.

### 2.2 Field measurements

Three 5.2‐ha observational plots, measuring 260 × 200 m each, were established in the summer of 2005 (Figure 1). All trees with diameters at breast height (dbh) ≥1 cm were measured and stem‐mapped. Remeasurements were conducted at regular intervals of approximately 5 years. The data used in this study were obtained in 2010 by the first remeasurement. The three plots are different in species composition and are referred to as a secondary conifer and broad‐leaved mixed forest (CBF), a secondary poplar and birch mixed forest (PBF), and a mixed Tilia and Korean pine forest (TKF). The disturbances in this region were the last recorded tree‐felling activities took place about 50 years ago. Table 1 presents the basic statistics of the three plots.

**Table 1:** Basic statistics of study plots

	Latitude	Longitude	Av. altitude (m)	No of trees	No of species	No of families
Conifer and broad‐leaved mixed forest	N42°20.907′	E128°7.988′	748	16,544	50	17
Poplar and birch mixed forest	N42°19.168′	E128°7.819′	899	29,309	64	20
Tilia and Korean pine forest	N42°13.684′	E128°4.573′	1,042	12,063	22	10

We recorded 16,544 living stems in CBF belonging to 17 families and 50 species, 29,309 living stems in PBF belonging to 20 families and 64 species, and 12,063 living individuals in TKF belonging to 10 families and 22 species. Details about the experimental plots are presented in Table 1.

### 2.3 Sampling design

To examine the scale effects on beta diversity and the underlying causes, we divided the surface of each 5.2‐ha plot, based on four grain designs with grain sizes ranging from 20 × 20 m (0.04 ha) up to 50 × 50 m (0.25 ha) by increments of 10 × 10 m. Table 2 shows the number of grains, decreasing with increasing grain size, and the total area for each sampling design.

**Table 2:** Specific information for sampling designs in each plot

Grain size	20 × 20 m	30 × 30 m	40 × 40 m	50 × 50 m
No of grains	130	48	30	20
Total area (ha)	5.2	4.32	4.8	5.0

Three of the four designs have total areas <5.2 ha because the grain widths are not exactly divisible into the total plot width.

### 2.4 Environmental and spatial variables

In 2010, two soil samples were taken from the top 20‐cm layer in each 20 × 20 m grain. These samples were used to quantify the following five variables: organic matter (%), soil pH, total nitrogen, phosphorus, and potassium (p.p.m.). The average value of these two samples was used to represent the soil nutrients of each 20 × 20 m grain. The soil rootable depth was also measured in each grain. In the summer of 2017, we measured the elevation of four vertexes for each 20 × 20 m grain. On this basis, four topographical variables were calculated for each grain including mean elevation, aspect, slope, and convexity. The corresponding six soil variables for the other three grain sizes were estimated by Kriging interpolation (Journel & Huijbregts, 1978), and a trend surface analysis was performed before interpolation. We also interpolated elevation values of four vertexes for each 30 × 30 m, 40 × 40 m, and 50 × 50 m grain, which were used to estimate the four topographical variables. The analysis was conducted in software ArcGIS (version 10.3), and interpolation process was explained in Appendix S1. The spatial variables were modeled using principal coordinates of neighbor matrices (PCNM; Borcard & Legendre, 2002; Borcard, Legendre, Avois‐Jacquet, & Tuomisto, 2008). The PCNM variables were generated for each grain design from the spectral decomposition of the spatial relationships among grains. Moran's eigenvectors were computed for each grain design, and eigen functions with positive Moran indices were then generated (Dray, Legendre, & Peres‐Neto, 2006).

Following Blanchet, Legendre, and Borcard (2008), forward selection by permutations of residuals at the 5% level of significance was applied to obtain the significant environmental parameters and PCNM vectors to serve as explanatory variables. The analysis was performed using R (version 3.1.1) with the packages “spacemakeR” (Dray et al., 2006) for spatial analyses and “packfor” (Dray, 2005) for the forward selection.

### 2.5 Statistical analysis

#### 2.5.1 Calculating beta diversity

At each sampling grain, we obtained an *n* × *p* (grains‐by‐species) data table *X* by counting the number of living individuals in every grain of each species. The element *x*_*ij*_ in table *X* represents the abundance of species *j* in grain *i*.

The Hellinger distance, as proposed by Legendre and Gallagher (2001), was used to measure the dissimilarity in the species composition between plot grains. The species abundance data transformed by the Hellinger distance can be analyzed directly by canonical redundancy analysis (RDA), which provided an easier and more informative way for beta diversity assessment. Each observed *x*_*ij*_ value was transformed using the following transformation (Legendre & Legendre, 2012):

$$y_{ij}=\sqrt{x_{ij}/\sum_{k=1}^{p}x_{ik}},$$

where *k* is the species index, and *p* refers to the number of species in a given grain with row and column indices *i* and *j*. This transformation was applied to each of the three study areas and each grid design to convert the observed community table *X* to a corresponding table *Y* = [*y*_*ij*_]. After the Hellinger transformation, beta diversity was calculated as the total variance of table *Y*:

$${\text{BD}}_{\mathrm{Total}}={\text{SS}}_{\mathrm{Total}}/\left(n-1\right),$$

where SS_Total_ is the total sum of all the squares, representing the total variance of the community composition. The index BD_Total_ is the unbiased form of the total variance, that is, beta diversity, which also expresses the average dissimilarity among grains.

Following Legendre's method, the total variance of species composition can be partitioned into local contributions to beta diversity (LCBD). LCBD is the ratio of the sum of the elements belonging to the same sampling units (SS_*i*_) and the total sum of all the squares:

$$\text{LCBD}={\text{SS}}_{i}/{\text{SS}}_{\mathrm{Total}}.$$

In other words, LCBD is the contribution of sampling units reflecting the degree of the uniqueness of the sampling grains in terms of community composition (Legendre & De Cáceres, 2013).

#### 2.5.2 Variation partitioning of species composition

Variation partitioning was performed by canonical redundancy analysis (RDA). This method is used to decompose the variation in species composition into fractions which may be explained by the environmental and spatial variables. According to Peres‐Neto, Pierre, Stéphane, and Daniel (2006), the variation in species composition can be partitioned into four components: the variation explained by pure environmental variables (fraction [*a*]), the variation explained by spatial variation of environmental variables (fraction [*b*]), the variation explained by pure space (fraction [*c*]), and unexplained variation (fraction [*d*]). As fraction [*b*] is a spatially induced variation that results in the response variable changing along with the spatial variation of environmental variables (Borcard, Gillet, & Legendre, 2011), we incorporated fraction [*a*] and [*b*] (fraction [*a* + *b*]) as the variation explained by environmental variables and interpreted fraction [*c*] as variation explained by a spatial process. The explanatory ability of each factor is expressed using the adjusted coefficients of determinations ($R_{\mathrm{adj}}^{2}$ ).

The calculations of beta diversity were carried out in R (version 3.1.1) using the “*beta.div*” function (Legendre & De Cáceres, 2013). Variation partitioning analyses were performed using the package “vegan” (Oksanen, Kindt, Legendre, & O'Hara, 2007).

## 3 RESULTS

### 3.1 Beta diversity and local contributions to beta diversity

**Figure 2:**
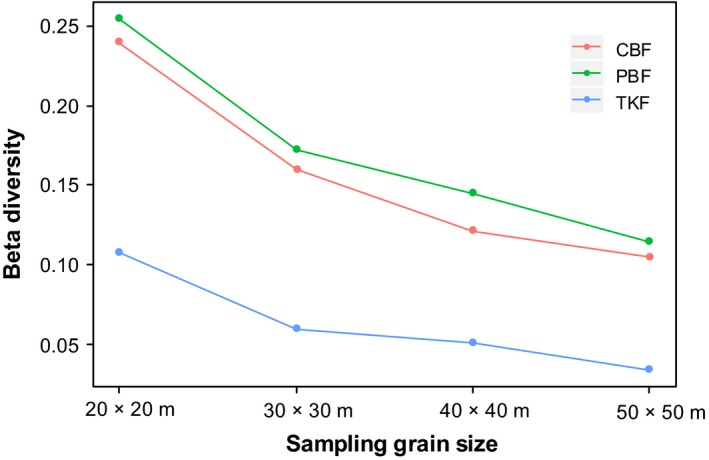
Relationship between grain size and beta diversity for the three study plots

As expected, beta diversity decreased with increasing grain size, as shown in Figure 2. The general form of the relationships is almost identical in the three study areas showing a consistent response to a change in grain area, with values ranging between 0.239 and 0.105 in CBF, between 0.254 and 0.114 in PBF, and between 0.108 and 0.034 in TKF.

The relationships in CBF and PBF (with 50, 64 species, respectively) are almost identical. There are only 22 species in TKF, and this low richness is associated with considerably low beta diversity values, although the shape of the TKF curve is similar to that of that of the CBF and PBF study areas.

**Figure 3:**
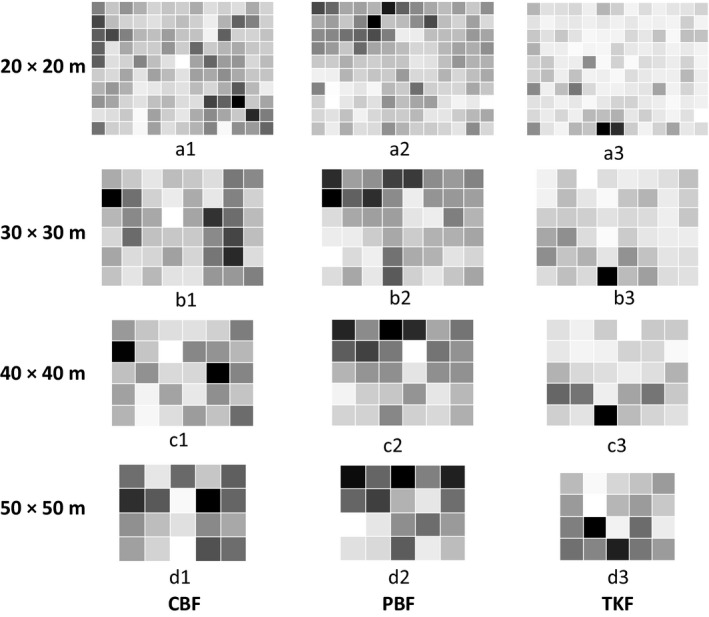
The spatial distribution of local contribution to beta diversity for the three study areas. The shading from light to dark shows that local contributions to beta diversity values increases from low to high

We mapped the LCBD values in Figure 3 which represent the degree of uniqueness of the sampling units in terms of species composition. As can been seen in Figure 3, compared with CBF and PBF, the LCBD values of most sampling grains in TKF were low represented by the light gray blocks, especially at the 20 × 20 m and 30 × 30 m scales. This was an indication that the species compositions of most sampling grains were similar. Only a few grains contained unique species compositions represented by the dark gray blocks. This result showed there was greater spatial variation in the species composition in CBF and PBF than in TKF, corresponding with the higher beta diversity values in CBF and PBF when compared with TKF.

### 3.2 Variation partitioning of beta diversity

**Figure 4:**
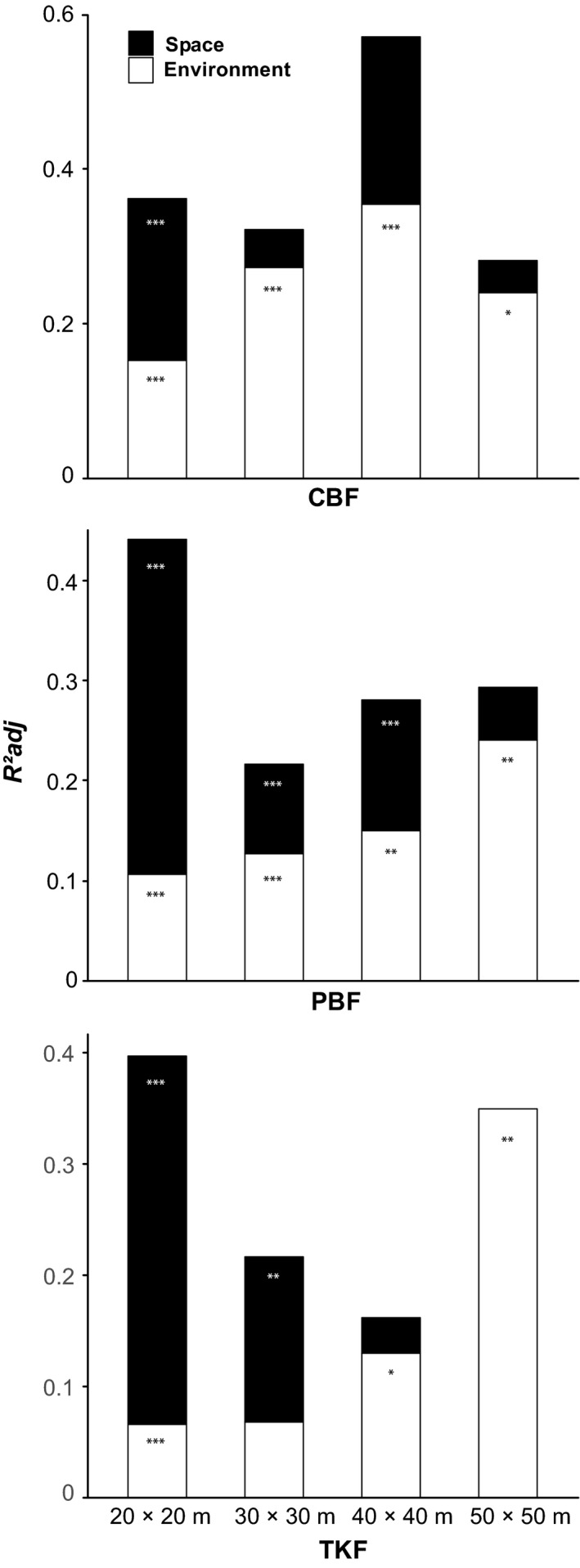
Results of the variation partitioning for the conifer and broad‐leaved mixed forest (top), poplar and birch mixed forest (middle), and Tilia and Korean pine forest (bottom) study areas. The white bars indicate environmental (fraction [a + b]), the black bars spatial effects (fraction [c]). The explanatory power of the two effects ($R_{\mathrm{adj}}^{2}$), soil and space, was established using 999 permutations at the 5% level of significance. ***p < .001, **p < .01, *p < .05

The results of the variation partitioning for the three study areas are presented in Figure 4. The histograms provided a visual summary of the relative importance of the spatial and environmental causes of beta diversity variation for the four sampling grains. Specifically, the best‐explained beta diversity variations across different sampling grains were found in CBF, where nearly 60% of the variation can be explained by environmental and spatial variables together at the 40 × 40 m scale. The variation partitioning results in the three study areas showed that both environmental and spatial variables have a significant influence on beta diversity variation. Our results also showed that environmental variables had the greatest explanatory power at the largest grain size across almost all three study areas (regardless of significance). The space variables, in turn, had the greatest explanatory power at the smallest (20 × 20 m) grain size. Comparatively speaking, the results of four sampling designs showed that environmental factors had a greater effect at grain sizes exceeding and including 30 × 30 m size. Spatial factors are more important at the 20 × 20 m scale. Meanwhile, we found an increasing relative importance of environmental effects with increasing sampling grain sizes. A striking increase in spatial effects was found in all three plots at 20 × 20 m scale. But the spatial effects were constantly weak at the remaining sampling grains and showed no clear trend with increasing sampling grain size. The number of alternative grains is rather low in the three largest grain sizes (48, 30 and 20 grains) which may have caused the somewhat inconsistent and insignificant results.

## 4 DISCUSSION

The decrease in beta diversity with increasing grain size reflected decrease in the compositional dissimilarity between sampling units (Figure 2). This result was consistent with the expectation that if spatial extent is fixed and sampling grain is allowed to vary, then beta diversity will decrease monotonically (Barton et al., 2013). In this study, the spatial extent was limited by 5.2‐ha (260 × 200 m) area, when grain size increased, a greater portion of the community was captured, and the similarity of the species compositions between the units increased. The results of this study resembled those presented by De Cáceres et al. (2012) who used some large study areas in different regions and grain sizes up to 100 × 100 m. In addition, in some previous studies, the sampling grain size was fixed, but the total study area varied (see for example Gering, Crist, & Veech, 2003; Lira‐Noriega, Soberón, Nakazawa, & Peterson, 2007). As expected, the beta diversity increased with increasing spatial extent of the study areas. These results show much variation of the relationship between beta diversity and spatial scale. Studies with different sampling design revealed different patterns and ecological mechanisms. A limitation of our study is that we only present the beta scaling relationship at local scales. Further researches are required to assess the effects of grain size on beta diversity at regional and even continental scales.

The spatial distribution of the local contributions to beta diversity showed that there was more compositional variation in CBF and PBF than in TKF. This result probably indicated that beta diversity was affected by differences in species richness. The greater the number of species in a plot, the greater is the possibility that the species composition varies among sampling units. And differences in composition variation (i.e., beta diversity) often obviously found along great environmental gradients which mainly caused by changes in the size of species pools (Kraft et al., 2011).

By partitioning beta diversity into different components, an attempt was made to investigate the underlying causes of the dissimilarity between sampling units, expressed in terms of beta diversity. The amount of explained variation of beta diversity may indicate how the forest community is affected by two underlying ecological processes, usually referred to as environmental filtering and dispersal limitation.

Environmental filtering is related to specific resource requirements that determine which species can survive and develop in a particular habitat. The increasing effect of environmental (soil and topography) variables in the three study plots as shown by the white bars in Figure 4 indicates that the environmental effect explains more of the variation at greater grain sizes and that species aggregate by the filtering effect of the soil and topographical properties. Habitat heterogeneity captured more spatial variation of the species composition with increasing grain size. In each of the three study areas, the small grains were not able to capture species specialization to environment as only about 10% of the variation is explained by environmental variables in the small cells. These results are consistent with those presented by De Cáceres et al. (2012). Dispersal limitation reflects the autocorrelation in the spatial structure created by purely spatial effects, which is independent of environmental factors. In each of the three study areas, dispersal limitation (the black bars in Figure 4) was greatest at the 20 × 20 m grain size. We may assume that in all three observational plots there is a high proportion of dispersal‐limited species carrying heavy seeds and propagating by gravity (e.g., *P. koraiensis, Q. mongolica, and Corylus mandschurica*). Also, species carrying light seeds (e.g., *Acer mono*;* Fraxinus mandschrica*;* T. amurensis*) may not be able to regenerate at greater distances as a result of shading and obstruction effects in the dense forests where canopies are closed and shading is usually severe. Species aggregation is especially conspicuous at the 20 × 20 m scale. It is, however, possible that a proportion of the spatial effect (black bars) is not due to dispersal limitation, but could perhaps be explained by other spatially structured environmental factors, such as availability of soil water, radiation, or microorganisms, which were not assessed. In this case, the results would overestimate the spatial effects and underestimate the environmental effects.

The relative importance of environmental and spatial processes has been the subject of several previous studies. Myers et al. (2013) found that environmental variables have a greater impact on beta diversity variation in temperate forests, while spatial effects explain a larger proportion of that variation in tropical forests. Qiao et al. (2015) reported that, in Badagongshan (a subtropical forest), topography and soil together explained 27% of beta diversity. This value is slightly less than the 34% explained by the spatial variables. In temperate forests, numerous studies claimed that environmental variables had a greater effect on beta diversity when compared with spatial effects (Gilbert & Lechowicz, 2004; Myers et al., 2013; Qian & Ricklefs, 2007; Wang, Fang, Tang, & Shi, 2012). All these studies are based on only one sampling grain to explain the causes of beta diversity. Our study which includes the important scale effects has shown that the underlying causes may be significant different, in a same region, depending on grain size. For example, spatial effects are dominant in the 20 × 20 m grain size, while environment is more important in the 50 × 50 m grain size (Figure 4). Similar results were presented by De Cáceres et al. (2012), which confirmed the importance of investigating scale effects.

The unexplained proportion of beta diversity variation may be related to unmeasured environmental variables, such as radiation and moisture. When the uncertainties are spatially structured but which is not explained by the measured environmental variables, studies would possibly overestimate the spatial effects and underestimate environmental effects (Qiao et al., 2015). Future studies should therefore collect as many environmental variables as possible. The unexplained portion of beta diversity variation may also relate to local stochastic processes (De Cáceres et al., 2012), such as death and recruitment. These processes create randomly different compositions of the sampling units (Alonso, Etienne, & McKane, 2006). When the number of individuals in the sampling unit is small (i.e., at small sampling scales or in plots with few individuals per unit area), this difference is more evident. It may also be evident in plots with higher species richness, because more species can be excluded by local ecological drift (De Cáceres et al., 2012). Thus, beta diversity is a result of complex process. The greater portion of unexplained variation does not necessarily indicate a greater importance of death and recruitment. But it is useful to capture differences in local beta diversity caused by some stochastic factors and processes that are hard to be quantified.

Our study examined the scale dependence of beta diversity and its underlying causes. For future studies, we emphasize the importance of choosing proper scales. When sampling grains are too small, there is a problem of undersampling. Because if the average number of trees per sampling unit is clearly less than the total number of species, many species for which the environmental conditions within a unit would be suitable will be absent simply because too few individuals were sampled. While at large grain sizes, the number of trees per unit usually exceed the total number of species of the entire plot. So if a species is absent, there is a bigger chance that this is not only a sampling artifact but also reflects something of ecological relevance. This may, for example, indicate that the differences among grain sizes would be not only due to the differences in how ecological processes function, but also may be due to the effects of undersampling at small scales. What is more, when sampling grain is smaller than the grains at which the environmental variables are sampled, interpolation to a smaller scale would increase the uncertainty of environmental variations and may result in incorrect variation partitioning. Conversely, when sampling grains are too big, the number of samples may be too small which affects the accuracy. Additional research is therefore needed to assess the ecological properties and mechanical causes of beta diversity of local forest communities.

## 5 CONCLUSION

Two hypotheses were presented in this study: (i) beta diversity of tree species decreases with increasing spatial scale within the same region and (ii) beta diversity is the result of different underlying causes at different sampling grains. It is shown that beta diversity variation is a complex response to environmental heterogeneity, species dispersal ability, and possibly some stochastic processes. Both beta diversity and its causes are affected by the sampling scales. The explained beta diversity variation across scales in all three plots showed that the effect of environmental filtering is more importance at greater grain sizes, whereas dispersal limitation is more important at small grain sizes especially effective at the 20 × 20 m scale. The effect of sampling scale on beta diversity and its underlying causes is considerable. Therefore, we conclude that, in future studies on beta diversity, the sampling scale is critical to identifying the different spatial and environmental drivers of species composition.

## AUTHOR CONTRIBUTION

Fan X.H. and Zhang C.Y. designed the experiment. Tan L.Z. and Fan C.Y. collected the data and conducted the statistical analysis. Tan L.Z. and K. v. G. interpreted the results and wrote the manuscript. All authors discussed the results and commented on the manuscript.

## CONFLICT OF INTEREST

None declared.
