# Supplementary material for: How beta diversity and the underlying causes vary with sampling scales in the Changbai mountain forests
Source: Ecol Evol. 2017 Oct 24;7(23):10116–23. doi: 10.1002/ece3.3493 (PMC5723590; doi:10.1002/ece3.3493)
Supplement: Supplementary file 1 [file ECE3-7-10116-s001.docx]

**Interpolation of soil variables:** The soil variables used in this study were sampled in the center of each 20×20m grains represented by the red dots in Fig.1. The soil data used at the other three scales were obtained from Kriging interpolation. The total area for 30×30m, 40×40m and 50×50m scales doesn’t match the original plot, so we cut the redundant edge. The interpolation process was conducted in software ArcGIS by using ArcTool *Kriging Interpolation*, the soil data at 30×30m, 40×40m and 50×50m scales were represented by green, purple and orange dots in Fig.1.


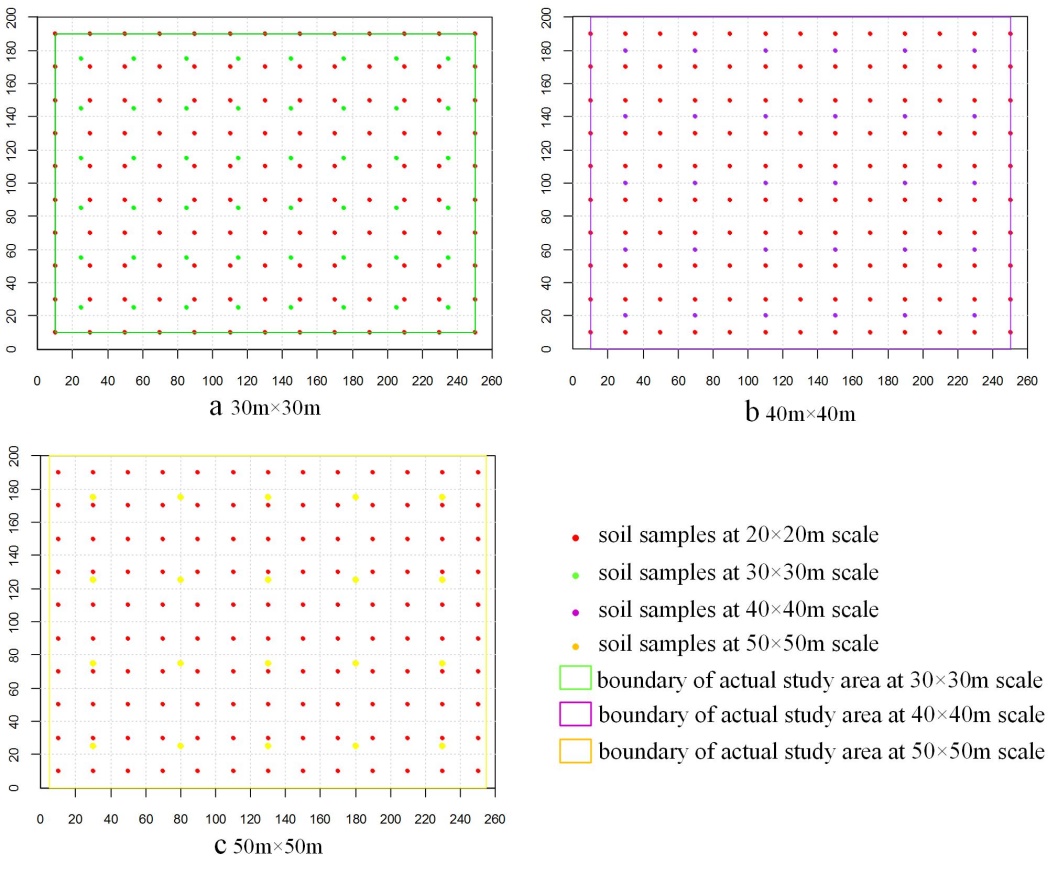


**Fig.1** The interpolation process of soil variables at 30×30m, 40×40m and 50×50m scales. The red dots represent the investigated soil data. The green, purple and orange dots represent interpolated data. The green, purple and orange rectangles represent the actual study area at 30×30m, 40×40m and 50×50m scales because the grain widths are not exactly divisible into the total plot width.

**Interpolation of topographical variables:** We measured the elevation values of the four vertexes for each 20×20m grain represented by the red dots in Fig.2. Analogously, elevation data used at 30×30m, 40×40m and 50×50m scales were obtained from Kriging interpolation, respectively represented by green, purple and orange dots in Fig.2. Four topographical variables, including mean elevation, slope, aspect and convexity, were calculated for each grain by using the elevation values of the four vertexes.


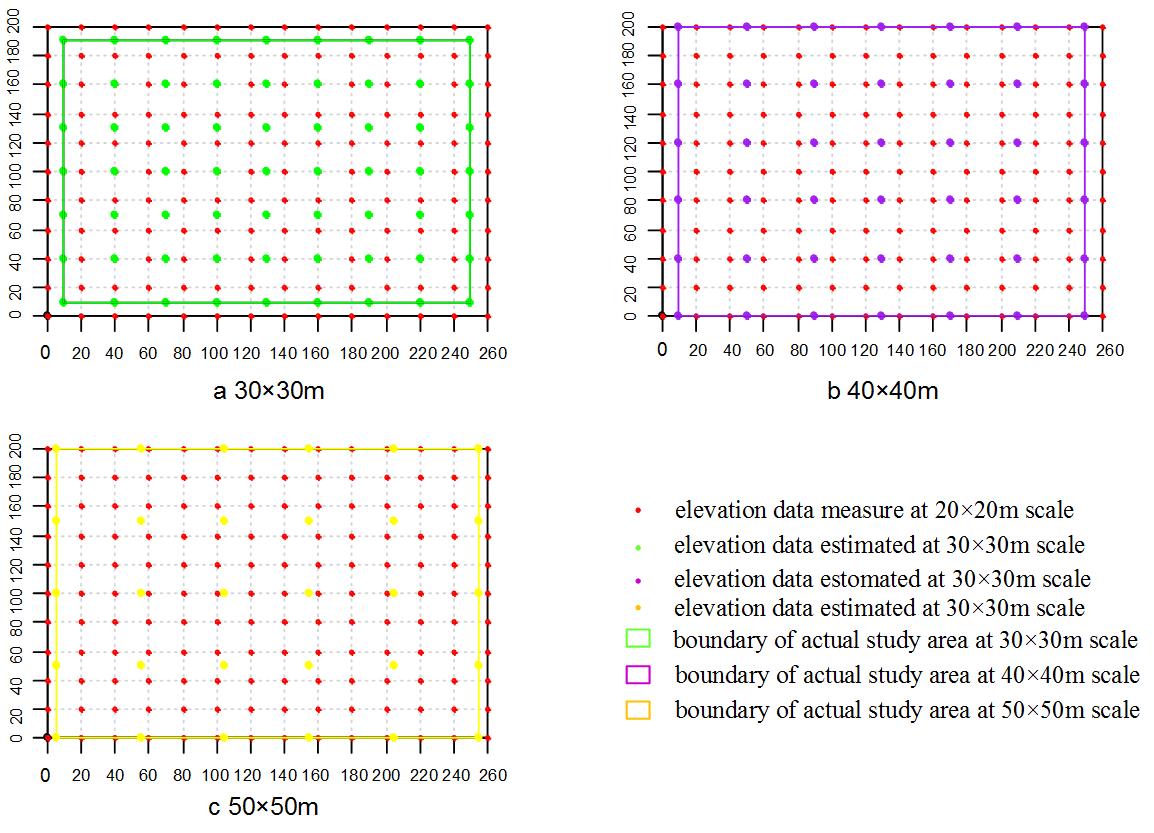


**Fig.2** The interpolation process of elevation data at 30×30m, 40×40m and 50×50m scales. The red dots represent the measured elevation data. The green, purple and orange dots represent interpolated data. The green, purple and orange rectangles represent the actual study area at 30×30m, 40×40m and 50×50m scales because the grain widths are not exactly divisible into the total plot width.
